# Scattering of NO Molecules from a Graphite Surface: Selectivity of the Rotational Excitation by Inelastic Collisions

**DOI:** 10.1002/cphc.202500213

**Published:** 2025-07-18

**Authors:** Maria Rutigliano, Fernando Pirani

**Affiliations:** ^1^ Istituto per la Scienza e Tecnologia dei Plasmi CNR(Consiglio Nazionale delle Ricerche) Via G. Amendola 122/D 70126 Bari Italy; ^2^ Dipartimento di Chimica Biologia e Biotecnologie Università di Perugia Via Elce di Sotto 8 06123 Perugia Italy; ^3^ Dipartimento di Ingegneria Civile ed Ambientale Università di Perugia Via G. Duranti 93 06125 Perugia Italy

**Keywords:** energetics, long‐range interactions, molecular dynamics, reaction mechanism, rotational distributions

## Abstract

The scattering of NO molecules from a graphite surface has been investigated using molecular dynamics simulations in conjunction with a new potential energy surface that properly accounts for the basic role of long‐range interactions on the collision dynamics. NO molecules impinge the surface in selected low‐medium roto‐vibrational states with collision energies ranging from subthermal to hyperthermal. The initial vibrational state is preserved in the triggered elementary processes while the molecules scatter into rotational states that follow well‐defined distributions. For medium‐low values of the initial rotational state, molecules are mostly excited, whereas for medium values, there is a slight quenching effect. Moreover, for medium‐high collision energies, the rotational distributions reveal a unique feature: two secondary peaks appear in the region of final high rotational states. This phenomenon relates to the configuration of the molecule as it approaches the surface. Moreover, when NO molecules at low collision energy collide with the O‐end facing the surface, the scattering predominantly occurs through a direct mechanism. Conversely, when the N‐end is directed toward the surface, the scattering involves an indirect mechanism, characterized by multiple bounces on the surface, accompanied by significant energy exchanges between the molecular internal degrees of freedom and the surface itself.

## Graphical abstract

Selectivity and propensity have been emphasized for NO molecules scattered from a graphite surface, employing molecular dynamics simulation alongside a new potential energy surface, including an appropriate treatment of the long‐range interaction, governing the formation of the precursor state, that then reacts with the surface. The interaction dynamics is fundamentally influenced by the end of the molecule facing the surface.

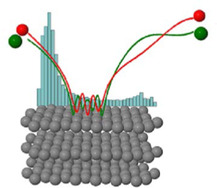

## 1 Introduction

The fate of gaseous molecules interacting with solid surfaces is mainly determined by elementary energy exchange processes promoted by collisions occurring at the gas‐surface interface. These processes can be conveniently highlighted and studied both from a theoretical‐computational and experimental side, through molecular dynamics (MD) simulations and molecular beams (MB) scattering, respectively.

Of particular relevance in MD simulations is the potential adopted to describe, in the complete range of relevant distances and molecular orientations, as accurately as possible, the interaction between the impinging molecule and the surface. Lately, we have highlighted the primary role played by weak (non‐covalent) long‐range forces and how an accurate description of them is necessary to obtain results in line with those provided by experimental measurements. In this context, we have introduced and used the Improved Lennard–Jones (ILJ) potential, recently formulated for the description of weak gas‐phase interactions^[^ ^1^ ^]^ and that we have proven also to be suitable for interactions that drive the collisions operating in the heterogeneous phase.^[^ ^2^ ^]^ The reliability of the ILJ formulation for the long‐range forces relies on the fact that it can adequately describe the precursor state formed by the incident species approaching the surface before reacting with them.

Recently, we adopted the ILJ potential to study in detail the scattering of small diatomic molecules from a graphite surface and we were able to highlight some propensities, selectivity, stereo‐dynamical effects due to rotational alignment and to determine the dispersion coefficients controlling the molecular attraction in the asymptotic region of separation distances (see ref. [3] and references therein). In particular, the investigation of CO scattering has shown how its heteronuclear feature induces in the scattering some peculiarities that do not occur in the case of homonuclear molecules.^[^ ^4^ ^]^ This latter point was the first stimulus to undertake the study of the interaction of NO with a graphite surface, taking into account that we have already investigated with the same methodology N_2_‐graphite^[^ ^5^ ^]^ and O_2_‐graphite^[^ ^6^ ^]^ homonuclear cases. In addition, the heterogeneous reaction between NO and CO catalyzed by a graphite surface is the most likely reaction leading to the chemical reduction of pollutant NO to non‐noxious N_2_.^[^ ^7^ ^]^ Hence, the availability of state‐resolved data for molecular processes at the surface becomes a need, since in some conditions the molecular internal energy content can improve the efficiency of a given reaction.^[^ ^8^ ^]^

The scattering of NO molecules from a graphite surface has been investigated both experimentally and theoretically since the 80s onwards by several research groups.

Rotational state populations and angular distribution of nitric oxide molecules scattered off graphite have been determined in pioneering MB experiments.^[^ ^9^, ^10^ ^]^ The authors of these studies argued for the primary role played by the energy exchanges occurring during the interaction and governing the collision dynamics. In particular, in ref. [10], a simple model based on transport properties is used to account for the obtained results, according to which the final rotational temperature is reached during the separation stage of the gas molecule from the surface.

The method of classical trajectories is used in ref. [11] to explain the results of experiments carried out a few years earlier.^[^ ^9^, ^10^ ^]^ In the adopted model, only a small part of the graphite surface is considered, while the used parameters account for the angle distributions measured in ref. [12]. What is found is that, for surface temperatures greater than the room temperature, molecules undergo rotational cooling and are scattered with a rotational temperature of 250 K, regardless of the considered surface. In ref. [13], Nyman et al. predicted a rotational rainbow in the final rotational distributions by adopting a statistical model to analyze the results obtained for the inelastic scattering of NO from graphite in experiments.^[^ ^9^, ^10^ ^]^ More recently, Greenwood and Koehler performed classical simulations to interpret the results that they obtained by using the Velocity Map Imaging technique, to study the dynamics of the interaction when a molecular beam of NO seeded in helium impinges on graphene supported on gold.^[^ ^14^, ^15^ ^]^ Although their findings may be conditioned by the fact that NO impacts graphene supported on gold, basically they obtained results similar to those previously found in experiments conducted on graphite^[^ ^9^, ^13^ ^]^ and in qualitative agreement with their experimental results. From the experiments, it also emerged that molecules are mainly scattered off through a direct mechanism, providing a decrease in their translational energy with partial conversion into rotational energy. In addition, evidence of the rainbow effect has been highlighted at high impact energies. Lately, in ref. [16], the interaction of NO on an oxygen‐functionalized highly oriented pyrolytic graphite (O‐HOPG) has been investigated through ab initio molecular dynamics simulations (AIMD), focusing on the formation of NO_2_ at the surface to control NO emissions. The authors of this work found that in NO scattering from an O‐HOPG surface, most of the collision energy goes into the collective motion of the carbon atoms in the topmost layer of the pyrolytic graphite surface and only a small fraction is transferred from translational to rotational molecular motion.

The target of the present study is to emphasize new selectivity accompanying the NO‐graphite scattering. Accordingly, we used a well‐consolidated computational setup based on chemical state‐to‐state MD simulations employing a semiclassical collisional method^[^ ^17^ ^]^ and adopting a new potential energy surface (PES), obtained by including the ILJ potential.^[^ ^1^ ^]^ The new PES is presented and discussed in comparison with other potential models and results available in the literature. The dispersion attraction coefficients, controlling the formation of the precursor state of all elementary processes involved, are also determined. The main aim of this study is to highlight the reaction mechanism and energy partitioning between the molecular internal degrees of freedom of impinging NO molecules in a well‐defined initial roto‐vibrational state for low surface temperature, where the energy exchanges can be easily highlighted. The reaction dynamics is also discussed by distinguishing the two possible configurations in which molecules can approach the surface, with N‐end or O‐end.

The article is organized as follows. The next Section provides basic details of the new PES, adopted to describe through MD simulations the interaction dynamics of NO colliding with a graphite surface. In the same section, the results for scattering probability and final roto‐vibrational distributions will be presented and discussed in conjunction with the assessment of the interaction mechanism and the anisotropic behavior of N‐end and O‐end in the scattering. Finally, after a brief summary on the main computational details, in the last Section, the main results are summarized and some conclusions are drawn.

## 2 Results and Discussion

### 2.1 Potential Energy Surface Determination

The PES for the NO‐graphite interaction has been obtained by using the same functional expression used in refs. [5, 6] as reported in Equation (1), adopting parameter values already used for the interactions of N, N_2_,^[^ ^5^ ^]^ and O, O_2_^[^ ^6^ ^]^ with the same graphite surface.

(1) $$V_{\text{int}}\left(r_{N-O}\;,\text{ R}\right)=\sum_{i=1}^{N}\sum_{k=1}^{2}V_{\text{NO}-\text{graphite}}\left(R_{\text{ik}}\right){\text{*f}}_{\text{sw}}\left(r_{N-O}\right)+\sum_{i=1}^{N}(V_{N-\text{graphite}}\left(R_{i}\right)+V_{O-\text{graphite}}\left(R_{i}\right))*\left(1-f_{\text{sw}}\left(r_{N-O}\right)\right)$$

with $f_{\text{sw}}\left(r_{N-O}\right)=-0.5*(\tanh\left(1.65{\text{ * r}}_{N-O}-4.8\right)-1.0)$.

The sum on *i* in the terms of Equation (1) runs on the *N* atoms of the surface model assumed in the simulations.^[^ ^5^, ^6^ ^]^ The analytical expressions of isolated N and O atoms interacting with graphite, $V_{N-\text{graphite}}$ and $V_{O-\text{graphite}}$ in the second term of Equation (1), are obtained as described in refs. [5, 6].

Instead, the first term in Equation (1) is obtained as a pure ILJ potential with parameters for N(in NO) and O(in NO) equal to those for N(in N_2_)^[^ ^5^ ^]^ and O(in O_2_).^[^ ^6^ ^]^ For completeness, these parameters are given again in **Table** **1**.

**Table 1:** Parameters for the ILJ used in the determination of PES in Equation (1). In the table, the values of C_6_ and C_3_ long‐range dispersion attraction coefficients are also reported.

	ε [meV]	R_m_ [Å]	β	C_6_ [meVÅ^6^]	C_3_ [meVÅ^3^]
N(NO)—C interaction	4.17	3.74	8.0	11 334	672
O(NO)—C interaction	4.41	3.64	8.0	10 228	606
N—C interaction	5.44	3.68	8.0	13 502	801
O—C interaction	4.62	3.62	8.0	10 327	617

The C_3_ coefficient for the interaction NO/graphite, determined by using the parameters of Table 1, is 1278 meVÅ^3^. This value is in good agreement with the value of 1252 meVÅ^3^, obtained by applying the analytical expression for the determination of the C_3_ coefficient for diatomic molecules interacting with a graphite surface proposed in ref. [3].

**Figure 1:**
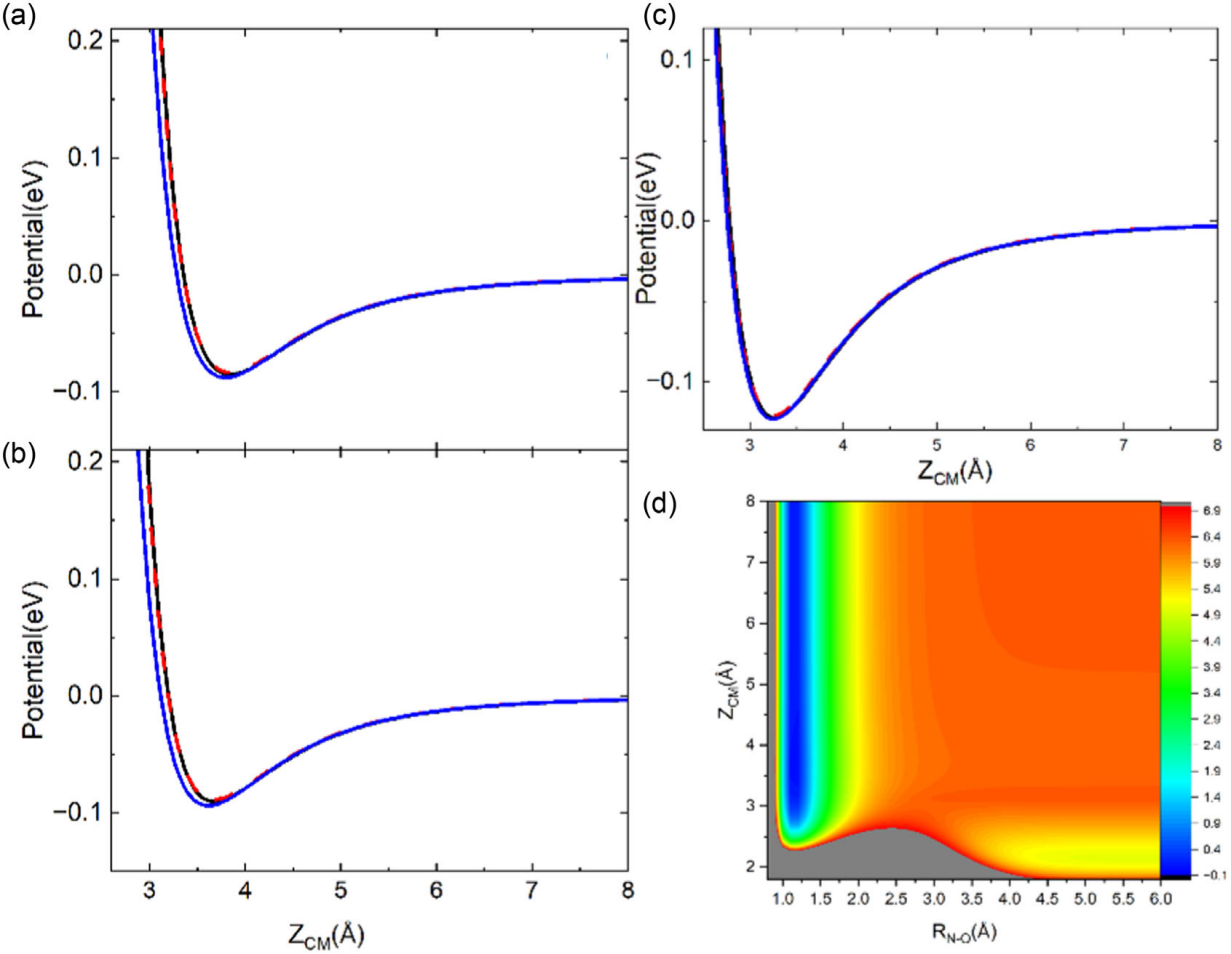
ILJ interaction potential for NO impinging perpendicular to the surface plane with (a) N‐end, (b) O‐end toward the surface, and (c) parallel to the *X*–*Z* plane on the three sites T (solid black line), B (dashed red line), and Hc (solid blue line). (d) 2D projection of complete PES for NO molecule impinging along a normal direction on top of a C atom in the surface with O‐end toward the surface.

The interaction potential obtained by using the parameters of Table 1 for NO/graphite interacting perpendicularly (with N‐end and O‐end toward the surface) and parallel with the molecular center of mass (CM) on top of a C atom on the surface (T site), in the middle of two neighboring C atoms (B site) and on the center of hexagonal unit cell (Hc), is reported in **Figure** **1**(a–c), respectively.

From the comparison between panels (a)–(c) of Figure 1, it emerges that among the considered molecular configurations, the physisorption is stronger when NO impacts the surface parallel to the XZ plane and, in such configuration, the adsorption energy (*E*_ad_) seems to be independent of the surface site where the molecule impacts. In particular, when the CM impacts the center of the graphite hexagonal cell (site Hc), *E*_ad_ is 123 meV while the binding distance is 3.25 Å. The interaction on the T site is slightly lower with *E*_ad_ equal to 122 meV at 3.28 Å, while the interaction is weaker on the B site, being *E*_ad_ equal to 120 meV for a distance of 3.25 Å. Instead, when the molecule collides perpendicular to the surface, we distinguished the case in which it impacts with N toward the surface from that in which it impacts with O. The adsorption energies (*E*_ad_) and the binding distance (*d*) are reported in **Table** **2**. In Figure 1(d), the 2D‐projection of complete PES (Equation (2)) for NO molecule impinging along the normal direction on top of a C atom in the surface with the O‐end toward the surface is shown.

**Table 2:** Values of E_ad_ and d for NO impinging perpendicular to the surface with N‐end and O‐end toward the surface on top of a C atom (T site), in the middle of two neighboring C atoms (B site) and on the center of the hexagonal unit cell (Hc site).

**Site**	N‐end toward the surface		O‐end toward the surface	
	E_ad_ [meV]	d [Å]	E_ad_ [meV]	d [Å]
**T**	85	3.23	90	3.15
**B**	83	3.23	88	3.13
**Hc**	88	3.18	94	3.08

From these findings, it emerges that there is not much difference between the various sites and different configurations, in agreement with what is reported in the literature.^[^ ^18^, ^19^ ^]^ However, among the available data, there are no unambiguous values according to the different software and methods used. In ref. [18], the binding energy and distance for the minimum energy configuration are 61 meV and 3.36 Å, respectively, by using the GGA (Generalized Gradient Approximation) method, while the LDA (Local‐Density Approximation) method with PWC functional gives the adsorption energy value of 82 meV. For NO colliding perpendicularly to the surface, our results are in line with those reported in ref. [19], in identifying as more stable the configuration with O‐end toward the surface, but there, the most favorable site is identified in B, while we found it to be the center of the hexagonal cell. Furthermore, in ref. [19], the distances at which adsorption takes place are longer than those found by us and are in the range 3.8–3.9 Å for N‐end molecule toward the surface and around 3.6 Å for O‐end molecule. However, it should be remarked that in ref. [20] calculations are made within the GGA approximation which can lead to overestimating bond distances and underestimating binding energies.^[^ ^21^ ^]^ In the same work, by using DFT calculations with Grimme's D2 method for the correction of dispersive terms, it is found *E*_ad_ = 140 meV at a distance of 3.26 Å for a configuration with the N atom, in NO, impinging on a C atom on the surface and O atom, in NO, pointing toward the center of the hexagon, with the molecule slightly tilted with respect to the normal to the plane. In ref. [22], by using BLYP functional with Grimme's D3 model for dispersion, an adsorption energy of 250 meV at a distance of 2.53 Å is obtained, but unfortunately, neither site nor configurations are specified. In ref. [23], for the molecule impinging parallel on the center of the hexagonal cell, a value of *E*_ad_ of 801 meV is obtained at a distance of 2.36 Å with a distance between N—O of 1.4 Å, as in the calculations the molecule and the substrate are completely relaxed. A higher value for *E*_ad_ (on the order of a few eV) can be found in ref. [20] for all sites and configurations with bond distances between 2.8 and 3.0 Å. The authors of ref. [24], using DFT calculations as implemented in CASTEP, obtained for the adsorption energy a value of 300 meV for a distance of 2.43 Å for the minimum configuration of ref. [19]. Instead, the authors of ref. [25] using again DFT calculations as implemented in CASTEP, find a binding energy of 190 meV for a distance of 2.67 Å and with the nitrogen atom toward the surface. For the reader's benefit and for a quick comparison with our results reported in Table 2, **Table** **3** provides a summary of the results cited in the previous lines for the adsorption energies and distances of NO on graphene, although the adopted calculation methods are different.

**Table 3:** Summary of adsorption energies and distances taken from the literature.

	Substrate material	Stable configuration	*E*_ad_ [meV]	*d* [Å]
Ref. [18]	Graphene	NO parallel slightly tilted with the N atom on C atom	61 (GGA) 82 (LDA)	3.36 (GGA)
Ref. [19]	Graphene	site B	140 (DFT‐D2)	3.26(DFT‐D2)
Ref. [20]	Graphene	NO parallel on B	29	3.76
Ref. [21]	Graphene	n.a.	250	2.53
Ref. [22]	Graphene	NO parallel on C	801	2.36
Ref. [24]	Graphene	NO parallel with N atom on C atom	300	2.43
Ref. [25]	Graphene	n.a.	190	2.67

From this overview of data available in the literature on the adsorption properties of NO on graphitic surfaces, despite some differences in the existing data, it emerges that the NO molecule is primarily physisorbed on the graphite surface. Furthermore, the results obtained for adsorption here are largely consistent, considering that it has already been shown that even DFT calculations with corrections for dispersive terms can yield less accurate values than those obtained using the ILJ potential.^[^ ^3^ ^]^

### 2.2 NO Scattering from Graphite

In the following, the most significant elementary processes triggered by NO molecules, impinging the graphite surface in different and well‐defined initial roto‐vibrational states (*j*_i_, *v*_i_) and collision energy (E_coll_), are reported and discussed. In the MD simulations, focused exclusively on the basic selectivity of molecular adsorption and scattering, the following initial roto‐vibrational states were considered: NO(0,0), NO(1,0), NO(0,5), NO(1,5), NO(10,0).

For the vibrational ground‐state and the lowest collision energies, the dominant process is molecular adsorption, which occurs with a probability of 0.8. However, this probability rapidly decreases as the collision energy increases, dropping to a value of 10^−2^ at *E*_coll_ = 0.1 eV. This finding agrees qualitatively with the results reported in refs. [14, 15]. As the collision energy and/or *v*_i_ increase, scattering becomes the only occurring surface process. It is worth noting that the anisotropy of the NO molecule leads to a higher probability of molecular adsorption at the lowest collision energy compared to corresponding processes involving oxygen and nitrogen molecules, with O_2_^[^ ^6^ ^]^ having a greater adsorption probability than N_2_.^[^ ^5^ ^]^ Moreover, NO molecules, impinging the graphite surface in the *v*_*i*_ values here considered, are scattered off in the same vibrational state with which they collide, as already foreseen for N_2_ and O_2_ scattered by the same surface.^[^ ^5^, ^6^ ^]^ This occurrence is in agreement with the findings of ref. [26], which established a high survival probability for the vibrational state and suggested a weak coupling of this motion with rotational and translational ones.

**Figure 2:**
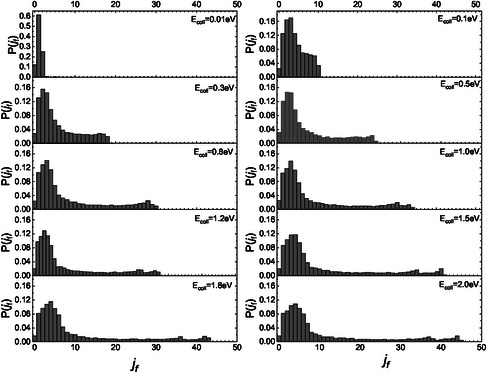
Complete final rotational distributions after the scattering achieved by NO(0,0) impinging on graphite at different collision energies.

The complete final rotational distributions for NO, resulting when the molecule impinges the graphite surface in the ground roto‐vibrational state, in the whole range of collision energies here investigated, are displayed in **Figure** **2**.

**Figure 3:**
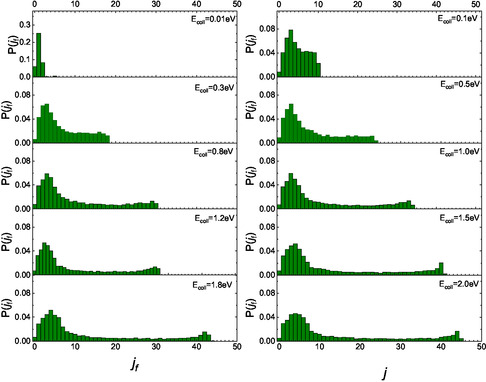
Partial final rotational distributions obtained for NO(0,0) molecules impinging with the N‐end toward the surface.

**Figure 4:**
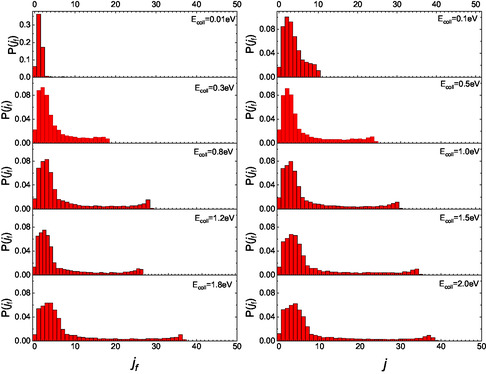
Same as Figure 3 but for NO molecules impinging with the O‐end toward the surface.

Figure 2 clearly shows that at *E*_coll_ = 0.01 eV, only the first three levels are populated. As the collision energy increases, the maximum populated *j*_f_ value also increases, which agrees with the findings of ref. [15]. This trend at the lowest energy significantly differs from what is observed for nitrogen and oxygen molecules impacting the same surface. In those cases, the distribution displays a bell‐shaped curve centered around *j*_f_ = 5, extending from *j_f_* = 0 to j_*f*_ = 15. For higher collisional energy values, the final distributions of NO(0,0) display a common trend, both qualitatively and quantitatively, similar to those of N_2_(0,0) and O_2_(0,0). However, in the case of NO, as the collisional energy increases, two secondary peaks appear at high values of j_*f*_. In contrast, only one secondary peak is observed for homonuclear molecules, N_2_ and O_2_.^[^ ^5^, ^6^ ^]^ To understand this trend, similarly to what was done in the case of the CO molecule,^[^ ^4^ ^]^ we sought to identify possible differences in the final rotational distributions for the two scenarios in which the molecule initially faces the surface with either its N‐end or O‐end. The partial final distributions for these two different initial configurations are presented in **Figure** **3** and **4**.

For E_coll_ greater than 0.1 eV, unlike what has been previously reported for CO,^[^ ^4^ ^]^ the configuration of the molecule, whether it impacts the surface with N‐end or O‐end, does not affect the appearance of a secondary peak at high values of *j*_*f*_. This combined behavior is responsible for the two secondary peaks observed at high *j*_*f*_ in the complete distributions shown in Figure 2. Moreover, the shift of the secondary peak to a higher *j*_*f*_ in the N‐end case, compared to the O‐end case, can be attributed to the differing “torque” experienced by the molecule during each gas‐surface collision. This torque selectivity results from the different masses of oxygen and nitrogen, as well as their respective positions relative to the center of mass of the NO molecule, which influences inelastic events. Therefore, what in refs. [14, 15] is observed and renamed as a “double rainbow,” manifesting at the highest kinetic energies analyzed, finds an explanation here in terms of the initial configuration with which the molecule impacts the surface. Additionally, the different behavior of nitric oxide compared to carbon monoxide can be attributed to both the variations in the potential corrugation and the role of the “torque.” It is worth highlighting that the rainbow effect in rotational distributions of scattered molecules has also been predicted in the past in purely statistical simulations.^[^ ^13^ ^]^

We believe that comparing the partial distributions in Figure 2 and 3 with those obtained for the corresponding homonuclear molecules could be useful. This comparison reveals that, at the lowest collisional energy, the distribution for NO, when the molecules collide with the N‐end, exhibits a behavior completely different from that obtained for the scattering of N_2_(0,0).^[^ ^5^ ^]^ Specifically, at *E*_coll_ = 0.01 eV, the distribution for N_2_ shows a bell‐shaped trend centered around *j*_f_ = 5 and extending up to *j*_f_ = 15, while for NO, only three levels are populated. Conversely, at higher energies and in low‐medium lying rotational levels range, distributions for NO are broader than those for N_2_. Additionally, it is noteworthy that, at medium‐high energies, the secondary maximum appears at lower *j*_f_ values for N_2_ than for NO. When we compare the distributions for O_2_ molecules^[^ ^6^ ^]^ with the partial distributions here obtained for incident NO with O‐end, we observe that, aside from the difference at *E*_coll_ = 0.01 eV, like to the findings for N‐end molecules in comparison with N_2_, the distributions exhibit a similar trend. However, the secondary peak in the region of high *j*_f_ for NO molecules appears at lower values than for O_2_ under the same dynamic conditions. Therefore, we can argue that the effect of the end furthest from the surface is to determine the position of the secondary maximum.

Moreover, the partial distributions for initially O‐end molecules, obtained in the case of CO scattering from the same surface,^[^ ^4^ ^]^ exhibit a trend very similar to that observed for NO. Thus, these results confirm the role of the end with which heteronuclear molecules impact the surface in determining, first, the fate of the collision event and then the low‐medium region of the final rotational distributions. However, this behavior closely relates to the very similar potential for NO and CO when molecules impact with O‐end toward the surface.

**Figure 5:**
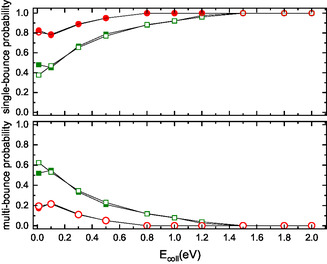
Single/multiple bounce probability as a function of E_coll_ for NO(0,0) (full symbols) and NO(0,5) (open symbols) molecules scattered from the graphite surface. Green and red symbols indicate molecules impinging with N‐end and O‐end toward the surface, respectively.

A thorough analysis of trajectories has revealed that, at medium‐high E_coll_, both N‐end and O‐end impinging molecules contribute equally to the scattering events. However, this is not the case at the lowest E_coll_ (0.01 eV) where the scattered molecules are primarily those with O‐end facing the surface. Furthermore, the scattering mechanism at this extremely low collision energy differs from that operating at higher collision energy values. This distinction is evident in **Figure** **5**, which reports, as a function of E_coll_, the probabilities of scattering due to single (direct mechanism) or multiple bounces (indirect mechanism) for impinging molecules initially colliding with either N‐end and O‐end facing the surface. The obtained results emphasize, at low E_coll_, the significant role of the indirect mechanism, the one promoting molecular adsorption, which selectively emerges for N‐end facing the surface.

Moreover, many of the considerations made above for *j*_i_ = 0 also apply to the case where *j*_i_ = 1, although some differences are evident. Specifically, the initial vibrational state again remains constant during the interaction. However, the final rotational distributions, while appearing very similar to those obtained for *j*_i_ = 0, show noteworthy differences upon closer examination, particularly for *E*_coll_ = 0.01 eV. The peak for *j*_f_ = 1 is reduced in height, making *j*_f_ = 2 more probable. Consequently, these two states have approximately the same probability (see Figure S1 in Supporting Information). This variation is attributed to the different ways in which molecules interact when colliding with the N‐end or O‐end, as displayed in the corresponding distributions (see Figure S2 and Figure S3 in Supporting Information). Indeed, for N‐end molecules, the probability of the single bounce mechanism slightly increases, while that of multibounce slightly decreases compared to the results for the roto‐vibrational ground‐state reported in Figure 5. Conversely, for O‐end molecules, the opposite trend is observed.

**Figure 6:**
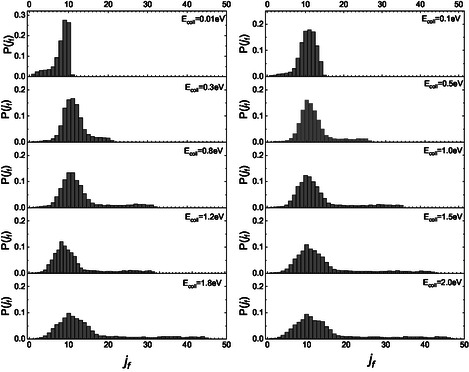
Complete final rotational distributions after the scattering achieved by NO(10,0) impinging on graphite at different collision energies.

As *v*_i_ is increased to 5, even at low E_coll_ values, the prevalent process becomes the scattering, a scenario different from that of molecules impinging with *v*_i_ = 0, where the adsorption process dominates at the lowest collision energy. For *v*_i_ = 5, the scattering process primarily involves NO molecules colliding with the N‐end facing the surface, contrary to what happens in the case of the vibrational ground state. A different behavior is also observed for the probability of single or multiple bounces, as can be inferred looking at Figure 5. This finding can be once again related to the position of the N atom relative to the molecule CM. The initial vibrational state is preserved while the rotational distributions display a pattern very similar to what is observed for *v*_i_ = 0 and *j*_i_ = 0,1. Specifically, the same considerations made in the case of NO in the vibrational ground‐state and in the two lowest rotational states also hold for *v*_i_ = 5, foreseeing for NO molecule what was found for O_2_ and N_2_ for low‐medium *v*_i_. Moreover, the final rotational distributions are primarily influenced by the initial rotational state and appear to be independent of the *v*_i_ value, as previously stated in ref. [11]. On the other hand, taking the colliding molecules in *v*_i_ = 0 and increasing the initial rotational state to *j*_*i*_ = 10, the molecular adsorption has yet a nonzero probability for the lowest collision energy, and the initial vibrational state is preserved, while the final rotational states yield distributions, as shown in **Figure** **6**, which peak around *j*_f_ = *j*_i_.

**Figure 7:**
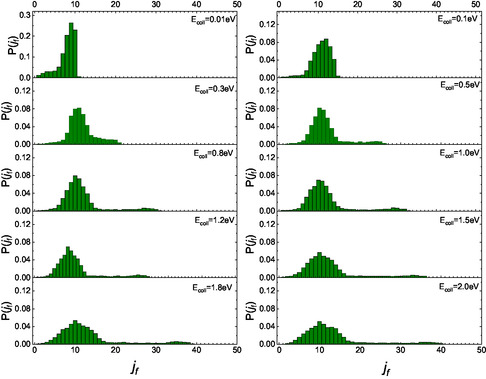
Partial final rotational distributions obtained for NO (10,0) molecules impinging with the N‐end toward the surface.

**Figure 8:**
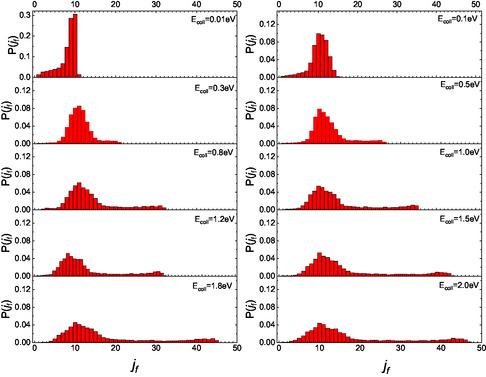
Same as Figure 7 but for NO molecules impinging with the O‐end toward the surface.

Analyzing the partial distributions for scattered molecules that impact the surface with either N‐end and O‐end facing down (**Figure** **7** and **8**), we find that, at the lowest E_coll_ value, molecules impinging with N‐end produce rotational distributions peaking at *j*_f_ = 9, which leads to a quenching effect on the rotational state of the NO impinging molecule. As the collision energy increases, for molecules that strike with N‐end, the peak at high *j*_f_ values nearly disappears. In contrast, for molecules arriving with O‐end toward the surface, this peak is significantly reduced but still remains apparent. Consequently, the peak at high *j*_*f*_ in the overall distributions of Figure 6 is also affected accordingly.

**Figure 9:**
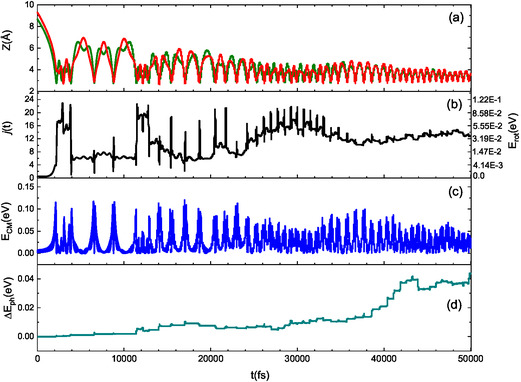
Typical trajectory for NO(0,0) impinging graphite (*T*_S_ = 100 K) with *E*_coll_ = 0.01 eV. At the beginning (large Z), NO shows the N‐end (green line) toward the surface, while the O‐end (red line) is facing the surface in proximity of closer separation distances (short Z). At the end of the trajectory, NO remains adsorbed on the surface rotationally excited *j*_f_ = 13. For the sake of clarity, on the right *y*‐axis of plot in the panel (b), the rotational energy, corresponding to the rotational level on the left *y*‐axis, is displayed.

**Figure 10:**
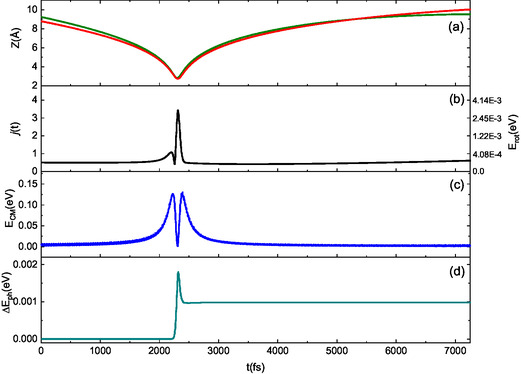
Typical trajectory for direct scattering of NO(0,0) impinging with O‐end (red line in panel (a)) toward the surface. The molecule is scattered with a helicopter‐like motion and with a small rotational excitation in the final state (1,0). *E*_coll_ = 0.01 eV and *T_S_* = 100 K. For the sake of clarity, on the right *y*‐axis of plot in the panel (b), the rotational energy, corresponding to the rotational level on the left *y*‐axis, is displayed.

**Figure 11:**
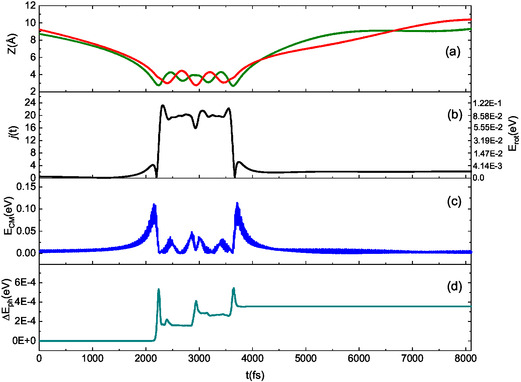
Typical trajectory for NO(0,0) impinging the surface with N‐end (green line in panel (a)) and scattered with a cartwheel‐like motion in the final state (2,0). *E*_coll_ = 0.01 eV and *T_S_* = 100 K. For the sake of clarity, on the right *y*‐axis of plot in the panel (b), the rotational energy, corresponding to the rotational level on the left *y*‐axis, is displayed.

We analyzed several trajectories in detail to better understand the mechanisms behind the basic selectivity of collision events, which arise from interaction anisotropy. Our analysis reveals that across the entire range of collision energies considered, the dynamics of interaction is primarily influenced by the motion of the oxygen atom in the NO molecule. To illustrate this, we will focus on the lowest collision energy, where the energy exchange mechanism is most active, and present three typical trajectories that exemplify the key aspects of the interaction dynamics. In **Figures** **9**, **10**, **11**, for collisional events (triggered by N‐end and O‐end molecules) ending with molecular adsorption and scattering, we report the time evolution of Z coordinate of N (green line) and O (red line) in NO (panel (a), CM translational energy (panel (b)), rotational state (panel (c)), and net energy exchange with the surface phonons (panel (d)).

Molecular adsorption typically occurs through a multibounce process and following a gradual damping of the motion. When the molecule, during the trajectory evolution, takes a configuration with the O atom facing and close to the surface, the adsorption probability is slightly higher than that for molecules approaching with the N‐end. This behavior, shown in Figure 9, as usual, can be attributable both to the greater mass of oxygen relative to nitrogen, which results in a different torque effect, as well as to the corrugation of the potential. From Figure 9, an interplay between the CM translational energy (shown in panel (c)) and the rotational number (shown in panel (b)) throughout the trajectory evolution also emerges. The energy exchanged with the surface phonons also contributes to this energy interplay. In Figure 9(d), the difference between the energy transferred and the energy taken from the surface is presented. Since this value is positive and increasing, we can conclude that a significant amount of energy is being transferred to the surface. Importantly, this transferred energy exceeds that associated with the molecular rotational excitation (≈37 meV at the end of the trajectory), so favoring the adsorption.

Moreover, when NO molecules are reflected into the gas phase, the different behavior of N‐end and O‐end impinging molecules leads to distinct motions of the scattered molecules. Specifically, molecules that impact the surface with their O‐end facing it tend to move with a helicopter‐like motion, which they maintain even after the interaction with the surface. As a result, these trajectories typically involve a single bounce (see Figure 10). In this scenario, the net energy exchange between the molecules and the surface remains positive; although the amount is much smaller compared to the adsorption process, it is still greater than the energy associated with the rotational excitation, 0.4 meV, at the end of the trajectory.

Conversely, when NO approaches the surface with its N‐end, the nitrogen atom undergoes a rotation. This behavior is similar to when N_2_ molecules interact with the same surface.^[^ ^5^ ^]^ Consequently, even if the molecule strikes the surface nearly parallel to it, it departs from the interaction with a cartwheel‐like motion (see Figure 11). This leads to a considerable loss of rotational energy gained during the interaction, causing the NO molecule to leave the surface with a low *j*_f_. This contrasts with the behavior of N_2_ and O_2_ molecules scattered from a graphite surface, where those scattered with a cartwheel‐like motion typically achieve a medium‐high final rotational state.^[^ ^5^, ^6^ ^]^ In this scenario, the net energy contribution to the surface phonons is minimal, indicating an almost equal energy flux from and to the surface.

The occurrence of an energy transfer from translation to rotational excitation has also been foreseen in the direct scattering from gold‐supported graphene:^[^ ^14^ ^]^ there, most of the energy is transferred to the motion of the graphene lattice. The same behavior is also reported in ref. [16] for NO molecules scattered from an O‐HOPG surface, where a significant part of the NO molecule energy is transferred to the considered surface. Moreover, the fraction of transferred energy slightly decreases when the interaction of the impinging NO molecule occurs directly with the epoxy group on the surface. Our calculations carried out at *T*_S_ = 100 K indicate (see Figure 9, 10, 11) that only a part of the energy involved in the interaction is transferred to the surface, although this quantity is higher for the adsorption processes and scattering of O‐end impinging molecules. Instead, when considering the surface at room temperature and NO molecules impinging with collisional energy confined between 0.01 and 0.1 eV, corresponding to the range controlled by the room temperature conditions, the average value of the net energy contribution transferred to the surface exceeds the average value of the energy transferred to the translation motion of the molecules. The partial agreement of these results with the findings of refs. [14, 15, 16] can be attributed to several factors. First and foremost is the difference in surfaces; one study involves a simple layer of graphene on a gold support, while the other pertains to an O‐HOPG surface. Additionally, the difference in the interaction potential used in the simulations may also contribute to these discrepancies. In refs. [14, 15], a simple Lennard–Jones potential with combining Lorentz−Berthelot rules is employed to rationalize the experimental results. In contrast, ref. [16] utilizes AIMD simulations based on a potential derived at the DFT level that includes dispersion corrections. Thus, a consistent and accurate description of long‐range interactions is essential, as these interactions determine how molecules interact with the surface and the energy transfer between the molecule and the substrate.

### 2.3 Computational Details

The scattering of NO molecules in selected initial roto‐vibrational states (*j*_i_, *v*_i_) from a graphite surface has been investigated, as said, by exploiting MD simulations based on a semiclassical collisional method.^[^ ^17^ ^]^ The method has been described and used in several previous articles in which the interaction of molecules with different surfaces is studied (see for instance ref. [3] and references therein, refs. [27, 28, 29, 30]). Therefore, only the salient points of the methodology will be summarized here, referring the interested reader to the previous literature. The method assumes that the interaction dynamics can be described by solving the 3D Hamilton's equations of motion self‐consistently with the dynamics of the lattice phonons, determined by solving the Schrödinger equation of motion for a set of 3*N*‐6 harmonic oscillators perturbed by the external force exerted by the impinging molecule, *N* being the number of atoms in the considered lattice model. Here, we assume the same surface model of ref. [3] and consequently also the same distribution of phonon states.

Along the trajectory integration, the solution of state‐to‐state evolution dynamics for the internal molecular degrees of freedom is also carried out. The complete Hamiltonian for the diatomic molecule interacting with the surface is given by

(2) $$H=\frac{1}{2}\sum_{i}\frac{P_{i}^{2}}{m_{i}}+V(r_{N-O})+\Delta E_{\text{ph}}+V_{\text{eff}}\left(t,T_{S}\right)\;i=1,2$$

where *P*_*i*_ is the momentum of atom *i* having mass *m*_*i*_ in the NO molecule, and *V*(*r*_N−O_) is the intramolecular potential that binds NO, being r_N−O_ the distance between the N and O atoms in the gas‐phase NO molecule. Δ*E*_ph_ is the energy exchanged with the surface phonons.

*V*_eff_ is a mean‐field type potential, depending on time (t) and surface temperature (T_S_), obtained as the expectation value on the phonon wavefunction of the interaction potential between the impinging NO molecule and the surface, V_int_.

Therefore, *V*_int_ basically represents the PES determined in the first subsection of the previous section.

Finally, the interaction dynamics are obtained by integrating the Hamilton equations at fixed initial conditions. For each set of initial roto‐vibrational states and collision energy, we propagated and analyzed 15 000 trajectories. NO molecules approach the surface along the normal to the surface plane, so that θ = 0°, while the azimuthal angles ϕ is determined according to the initial coordinates of N and O atoms in the molecule chosen randomly in an aiming area on the surface, large enough and at the same time such as to prevent the edge effects during trajectory propagation. The integration step is 0.25 fs, and the accuracy required in the integration procedure is 10^−8^. The surface temperature is fixed at 100 K, both for easy identification of similarities and differences in the dynamics and products of the interaction in comparison with the results obtained for the other molecules interacting with graphite, mainly N_2_ and O_2_, and because, at low surface temperature, the energy exchanges are more evident. The *E*_coll_ values range between 0.01 and 2.0 eV. The adopted PES enables, as in refs. [5, 6] to predict the occurrence of the different surface processes, such as molecular adsorption, molecular dissociation, dissociative adsorption/desorption, and atomic desorption, that can occur in addition to the scattering. The criteria adopted to discriminate between the various reaction channels, as we aim to focus on the selectivity of molecular adsorption and molecular scattering, are the same as those adopted in the previously mentioned papers.

## 3 Conclusion

The interaction of the NO molecule in well‐defined roto‐vibrational states has been studied through MD simulations using a newly developed PES that includes long‐range interactions. The same methodology has recently been applied to characterize the scattering of N_2_ and O_2_ molecules from the same graphite surface. This enables us to consistently underline both the peculiarities of the scattering of NO from graphite and to emphasize the similarities and differences with the most analogous systems, such as N_2_ and O_2_. The primary differences observed in the final rotational distributions of NO molecules are associated with the behavior at the lowest collision energy and the appearance of two secondary maxima in the region of high final rotational states as the collision energy increases. These differences are primarily determined by the torque exerted on the molecule, which stems from its heteronuclear nature. This characteristic also affects the interaction dynamics based on whether the molecule approaches the surface with the O‐end or the N‐end. Specifically, NO molecules impinging with O‐end are mainly scattered through a direct mechanism. In contrast, those approaching with N‐end follow an indirect mechanism, which results from the realignment of the nitrogen atom in the NO molecule. This realignment leads to a propensity for the molecules to scatter off in a cartwheel‐like motion.

The results obtained from this study, in conjunction with previous experimental and theoretical investigations,^[^ ^14^, ^15^, ^16^ ^]^ contribute to understanding the selective role of long‐range molecule–surface interactions on the dynamics of basic elementary processes occurring at gas‐surface interfaces, a topic of significant scientific‐technological interest.

## Conflict of Interest

The authors declare no conflict of interest.

## Supporting information

Supplementary Material

## Data Availability

The data that support the findings of this study are available from the corresponding author upon reasonable request.
