# Supplementary material for: Scattering of NO Molecules from a Graphite Surface: Selectivity of the Rotational Excitation by Inelastic Collisions
Source: Chemphyschem. 2025 Jul 18;26(17):e202500213. doi: 10.1002/cphc.202500213 (PMC12447121; doi:10.1002/cphc.202500213)
Supplement: Supplementary file 1 — Supplementary Material [file CPHC-26-e202500213-s001.pdf]

Supporting Information

for

Scattering of NO molecules from a graphite surface: selectivity of the rotational excitation by inelastic collisions

Maria Rutigliano*,[a] and Fernando Pirani[b], [c]

[a] Dr. M. Rutigliano
Istituto per la Scienza e Tecnologia dei Plasmi
CNR(Consiglio Nazionale delle Ricerche)
Via G. Amendola 122/D, 70126 Bari, Italy
E-mail: maria.rutigliano@cnr.it

[b] Prof. F. Pirani
Dipartimento di Chimica, Biologia e Biotecnologie
Università di Perugia
Via Elce di Sotto 8, 06123 Perugia, Italy

[c] Prof. F. Pirani
Dipartimento di Ingegneria Civile ed Ambientale
Università di Perugia
Via G. Duranti 93, 06125 Perugia, Italy

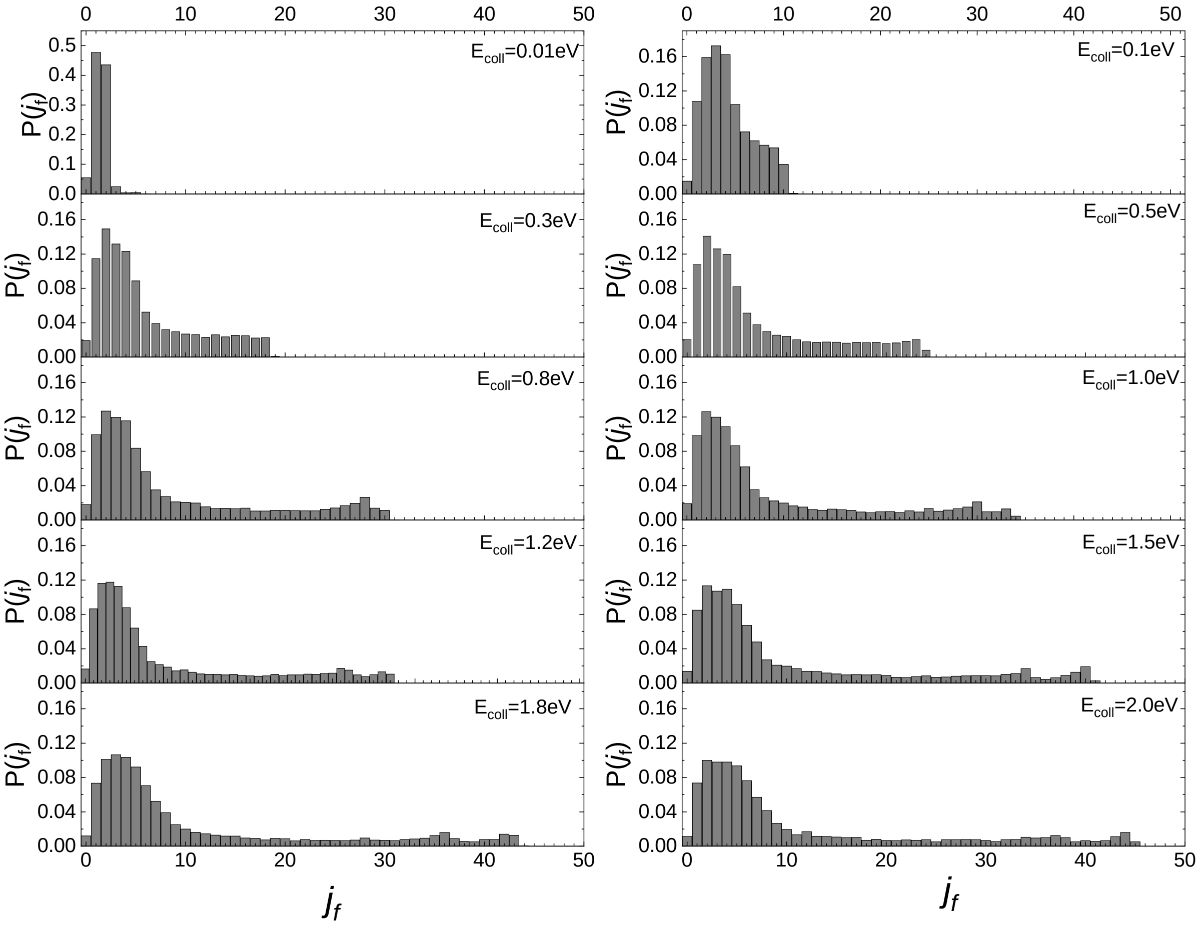

Figure S1: Complete final rotational distributions after the scattering achieved by NO(1,0) impinging on graphite at different collision energies.

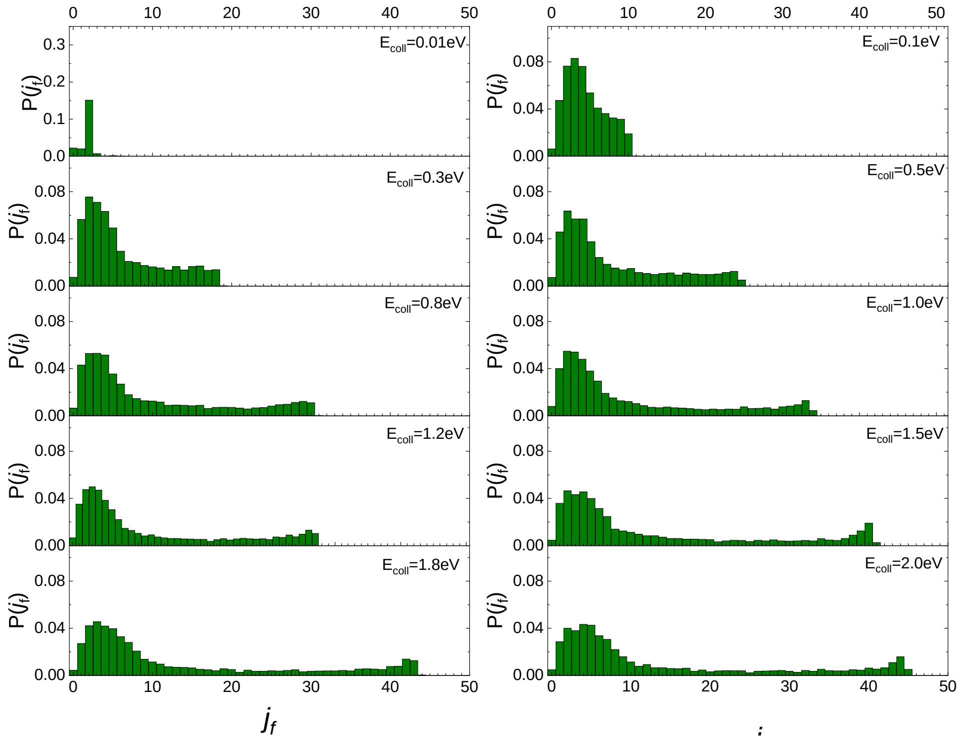

Figure S2: Partial final rotational distributions obtained for NO (1,0) molecules impinging with the N-end toward the surface.

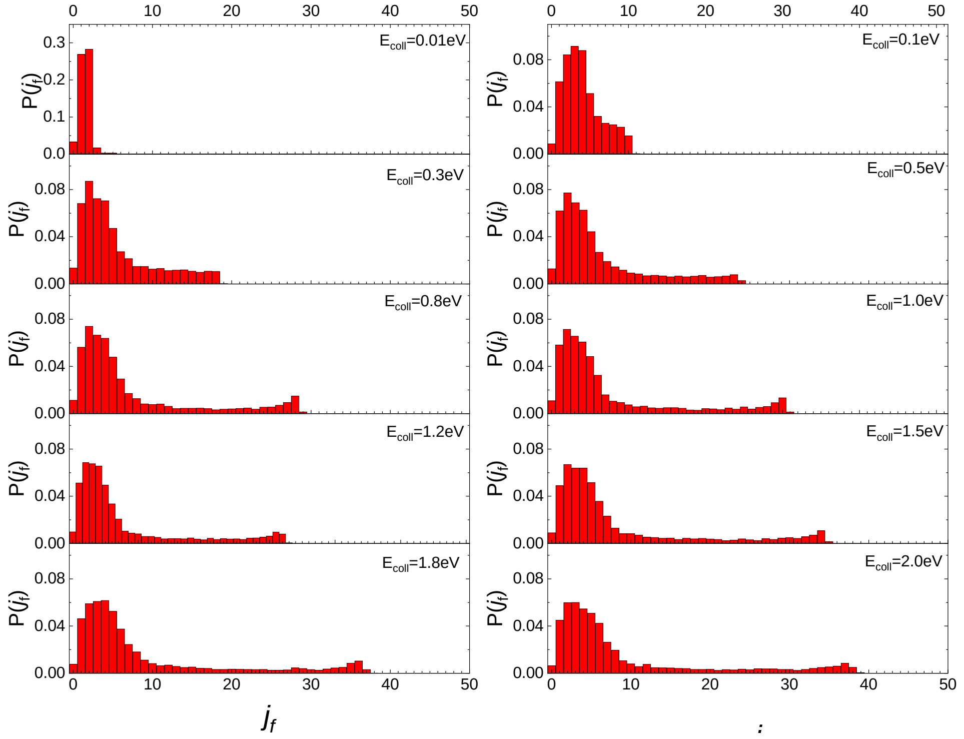

Figure S3: Same as Figure S2 but for NO molecules impinging with the O-end toward the surface.
